# HBV-related hepatocarcinogenesis: the role of signalling pathways and innovative ex vivo research models

**DOI:** 10.1186/s12885-019-5916-6

**Published:** 2019-07-18

**Authors:** Joseph Torresi, Bang Manh Tran, Dale Christiansen, Linda Earnest-Silveira, Renate Hilda Marianne Schwab, Elizabeth Vincan

**Affiliations:** 1 Department of Microbiology and Immunology, The Peter Doherty Institute for Infection and Immunity, University of Melbourne, Parkville, Victoria 3010 Australia; 2 The Peter Doherty Institute for Infection and Immunity, University of Melbourne, Parkville, Victoria 3010 Australia; 3 Victorian Infectious Diseases Reference Laboratory, The Peter Doherty Institute for Infection and Immunity, University of Melbourne, Parkville, Victoria 3010 Australia; 4 School of Pharmacy and Biomedical Sciences, Curtin University, Perth, WA 6845 Australia

**Keywords:** Hepatitis B virus, Liver cancer, Wnt signalling, Organoids, Cell cycle

## Abstract

**Background:**

Hepatitis B virus (HBV) is the leading cause of liver cancer, but the mechanisms by which HBV causes liver cancer are poorly understood and chemotherapeutic strategies to cure liver cancer are not available. A better understanding of how HBV requisitions cellular components in the liver will identify novel therapeutic targets for HBV associated hepatocellular carcinoma (HCC).

**Main body:**

The development of HCC involves deregulation in several cellular signalling pathways including Wnt/FZD/β-catenin, PI3K/Akt/mTOR, IRS1/IGF, and Ras/Raf/MAPK. HBV is known to dysregulate several hepatocyte pathways and cell cycle regulation resulting in HCC development. A number of these HBV induced changes are also mediated through the Wnt/FZD/β-catenin pathway. The lack of a suitable human liver model for the study of HBV has hampered research into understanding pathogenesis of HBV. Primary human hepatocytes provide one option; however, these cells are prone to losing their hepatic functionality and their ability to support HBV replication. Another approach involves induced-pluripotent stem (iPS) cell-derived hepatocytes. However, iPS technology relies on retroviruses or lentiviruses for effective gene delivery and pose the risk of activating a range of oncogenes. Liver organoids developed from patient-derived liver tissues provide a significant advance in HCC research. Liver organoids retain the characteristics of their original tissue, undergo unlimited expansion, can be differentiated into mature hepatocytes and are susceptible to natural infection with HBV.

**Conclusion:**

By utilizing new ex vivo techniques like liver organoids it will become possible to develop improved and personalized therapeutic approaches that will improve HCC outcomes and potentially lead to a cure for HBV.

## Background

Hepatitis B virus (HBV) is a major health concern in many regions of the world, where chronic carrier rates range from 10 to 20%. Despite the availability of a safe and effective vaccine, 5.2 million cases of acute infection were reported in the year 2000 and there are over 400 million chronic carriers globally. In Australia, 239,000 people are chronically infected, and there are an estimated 90,000 people who have not been diagnosed and are unaware of their infection. Liver cancer is the second most common cause of cancer death after lung cancer. More alarmingly, while the mortality rate for most cancers has decreased significantly over the last decade and is projected to continue this sliding trend over the next 20 years, liver cancer remains one of the common cancers with an increasing death rate. The main type of liver cancer is hepatocellular carcinoma (HCC). The mortality rate from HCC is projected to increase by ~ 40% by 2030 (Cancer UK). Over 90% of cases of liver cancer globally have a viral aetiology, and the vast majority of these is due to chronic hepatitis B infection [1]. In fact, the recent Global Burden of Disease study has highlighted that total deaths caused by viral hepatitis (including liver cancer) now exceed the number of deaths caused by tuberculosis, HIV/AIDS and malaria [2].

Although HBV is a known hepatocarcinogen, the precise mechanism by which it causes HCC is unknown and the optimal therapeutic regimens for the treatment of HBV associated HCC have not yet been established. Treatment options for HCC are limited and currently Sorafenib monotherapy is the standard of care for patients with advanced HCC. However, the overall survival of patients treated with sorafenib is disappointing with the median survival of less than 3 months [3]. There is an urgent need to develop new and more effective treatments for HCC. Mounting evidence from both in vitro and in vivo studies suggest that combination therapy could be an effective approach. For example, an additive and synergistic effect of targeting the Ras/Raf/MAPK pathway in combination with other pathways important in HCC proliferation such as Phosphatidylinositol-4,5-bisphosphate 3-kinase (PI3K)/AKT/ mammalian target of rapamycin (mTOR) and Wnt/β-catenin has been shown [4]. However, selecting the best, most appropriate and safest combinations, particularly for patients with HBV associated HCC and for clinical trials, remain a challenge.

## Main text

### HBV and hepatocarcinogenesis

**Fig. 1:**
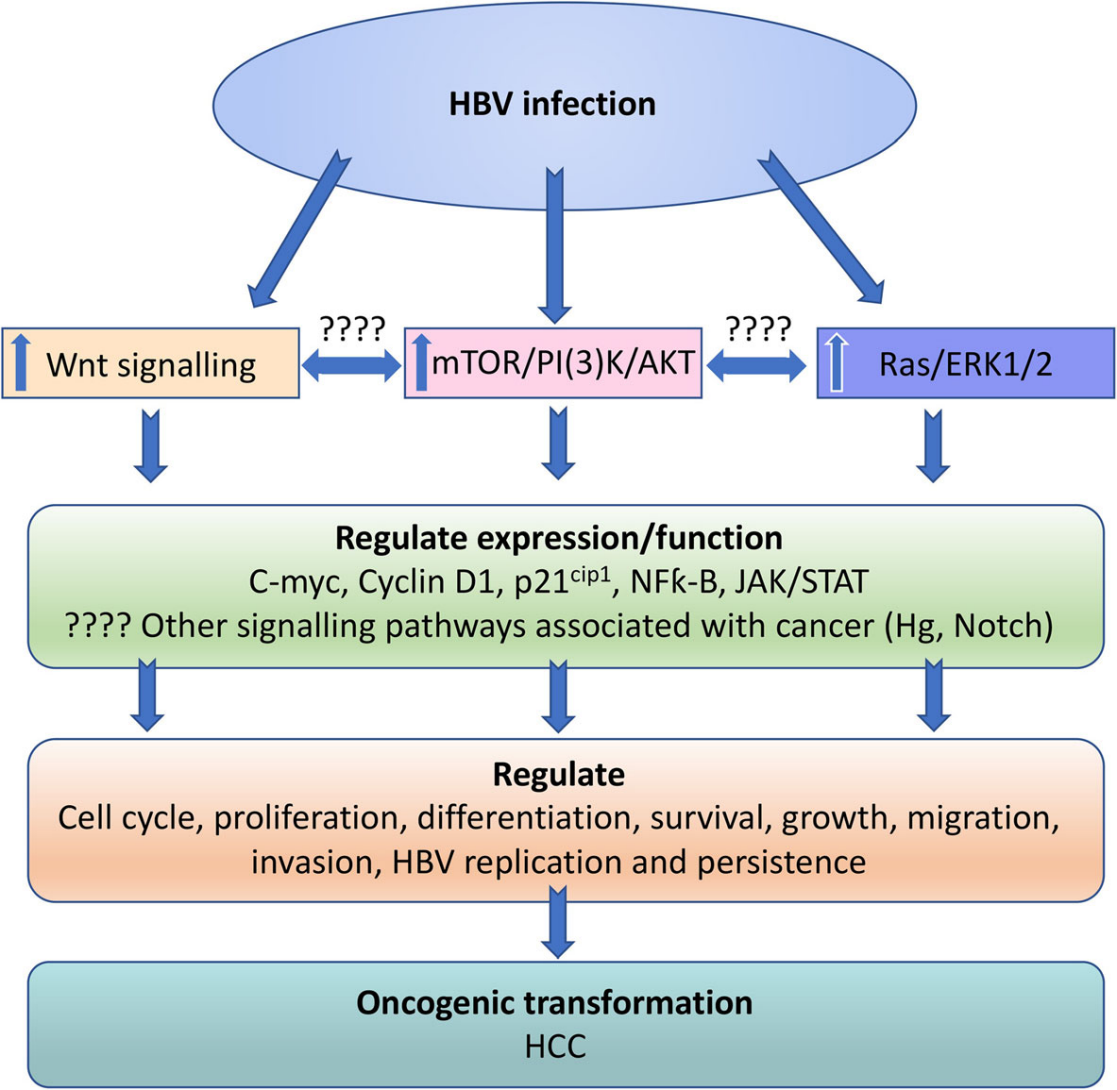
HBV associated HCC. HBV infection of hepatocytes is thought to impact on a number of cellular signalling pathways to regulate expression and function of genes that control cellular processes but also HBV replication and persistence, that ultimately leads to oncogenic transformation

The mechanism underlying the development of HBV associated HCC is multifactorial, linking changes within cell signalling pathways and cell cycle regulation, together with an inflammatory and cytokine reaction driven by antigen presenting cells in response to degradation products of apoptotic cells and viral antigens [5, 6]. Several separate signalling pathways are deregulated in HCC including the Wnt/FZD/β-catenin, PI3K/Akt/mTOR, insulin receptor substrate 1 (IRS1)/ insulin-like growth factor 1 (IGF), and the Ras/Raf/ mitogen-activated protein kinases (MAPK) pathway (Fig. 1). Inflammation that accompanies chronic hepatitis B infection of the liver is a strong factor in the development of HCC [5, 6] and currently available therapeutic strategies for HBV are only partially effective in reducing the risk of developing HCC [7]. The X protein of HBV (HBx) has also been shown to be an important promoter of hepatocellular transformation. We have previously shown that HBV results in dysregulation of several signal transduction pathways, cell cycle [8–10] and that the HBx protein contributes to HCC development through the upregulation of suppressor of cytokine signalling 3 (SOCS3) protein [11]. Similarly, the HBV large (LHBs) and middle (MHBs) envelope proteins have also been shown to contribute to hepatocarcinogenesis [12]. Furthermore, the effects of HBV on these signalling pathways are mediated through the Wnt/FZD/β-catenin pathway [6], a critical driver of HCC development. Inflammation that accompanies chronic hepatitis B infection of the liver is a strong factor in the development of HCC [5, 13–15] and currently available therapeutic strategies for HBV are only partially effective in reducing the risk of developing HCC [16]. Kupffer cells also appear to play a central role in driving the inflammatory responses that underlie HCC development [5, 15]. While interesting, these in vitro studies suffer from the inherent drawback that their relevance to HCC caused by expression of these proteins during the normal course of viral infection is unknown.

### HBV associated HCC, Wnt/FZD/β-catenin, PI3K/AKT, IN/IRS1, and Ras/ERK1,2 pathways

#### Wnt/FZD/β-catenin pathways

**Fig. 2:**
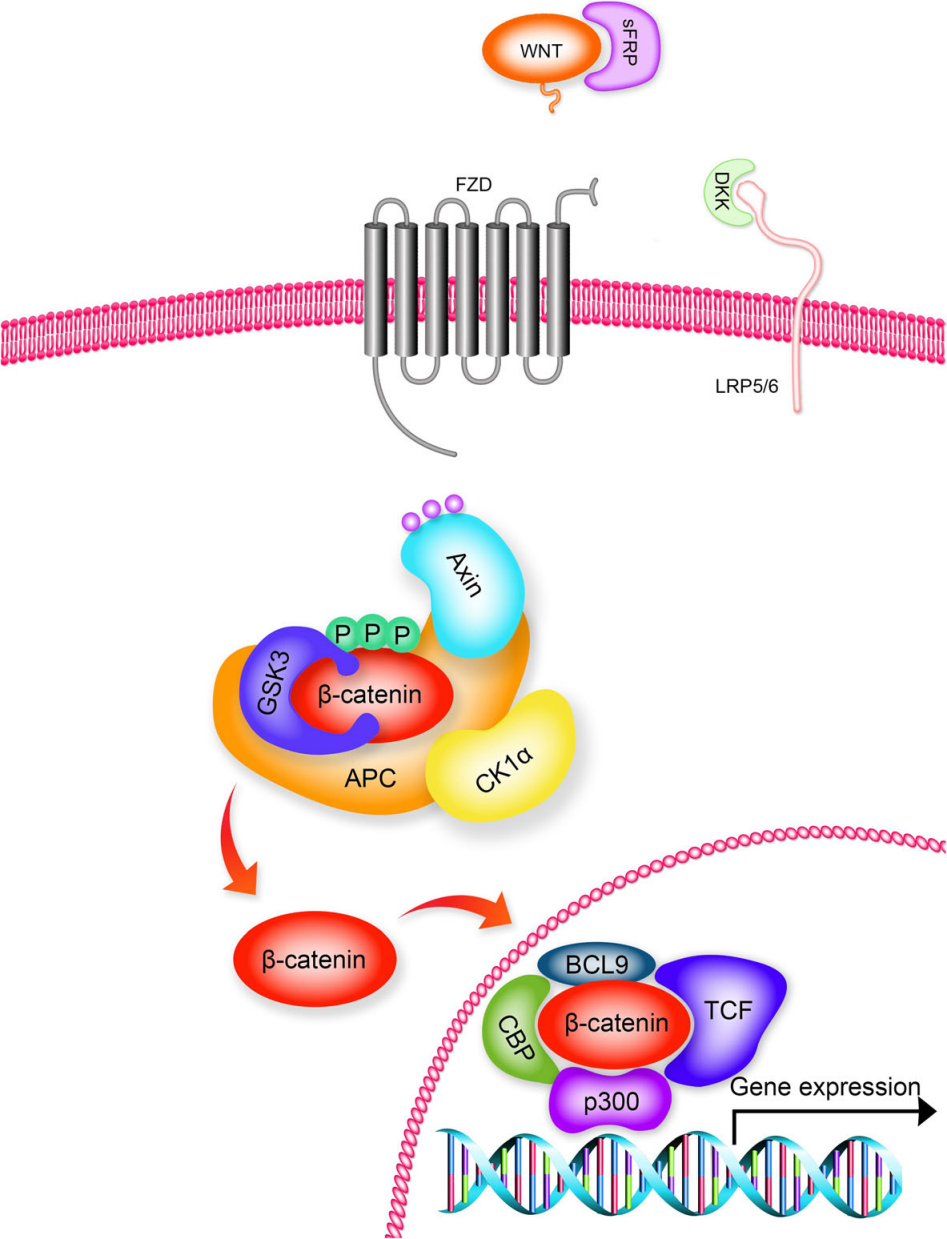
Wnt/β-catenin signal transduction pathway. Wnt binding to the FZD/LRP5/6 receptor complex leads to inhibition of GSK3 enzyme activity and the β-catenin destruction complex, which allows newly synthesized β-catenin to accumulate and translocate to the nucleus (orange arrows), where it binds with co-factors to form a transcriptionally active complex. Wnt signalling can be inhibited at the cell surface by various naturally occurring pathway inhibitors such as sFRPs and DKK, which bind to Wnt and LRP5/6 respectively. [secreted Frizzled Related Protein (sFRP); Frizzled (FZD); Dickkopf (DKK); Glycogen Synthase Kinase 3 (GSK3); Adenomatous Polyposis Coli (APC), Casein Kinase 1 (CK1)]

The Wnt cascade has emerged as a critical regulator of stem cells and activation of Wnt signalling has been associated with numerous cancers [17]. Wnt signalling is activated by the binding of one of the 19 mammalian extracellular soluble secreted Wnt ligands to single or multiple members of 10 mammalian Frizzled (FZD) receptors (Fig. 2). Binding of Wnt to FZD can lead to activation of the canonical β-catenin pathway [18, 19] or the non-canonical c-Jun N-terminal kinase (JNK) [20] and Ca^++^ pathways [21], however, here we limit our discussion to the canonical β-catenin pathway as its role is best characterised in context of liver and liver cancer.

**Fig. 3:**
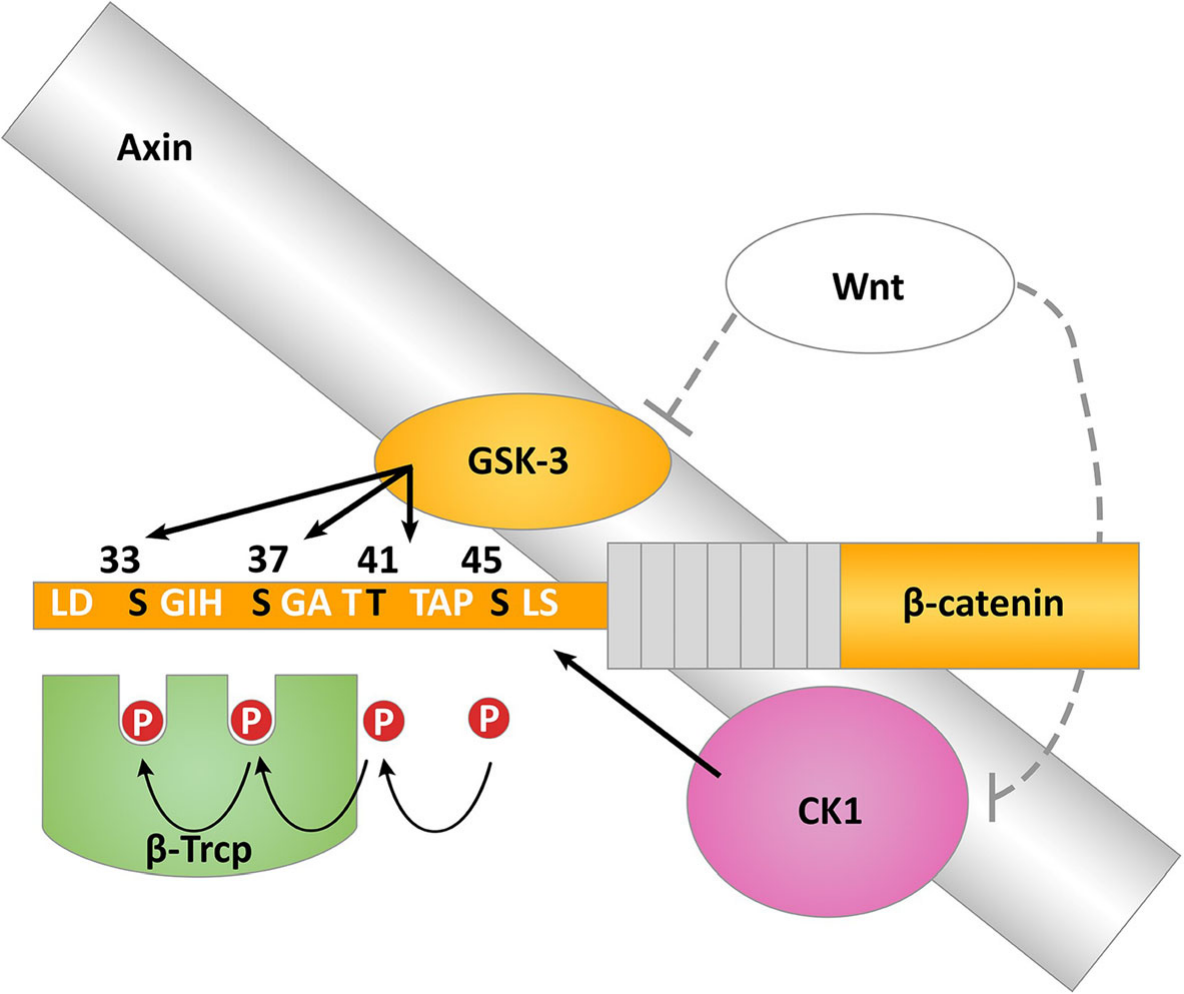
Targeted phosphorylation’s are required for ubiquitylation and degradation of β-catenin. Axin acts as scaffold that brings CK1, GSK3 and β-catenin in close proximity. CK1 initiates the process by phosphorylating Ser45 at the N-terminus of β-catenin, followed by sequential phosphorylation at Thr41, Ser37 and Ser33 by GSK3. β-Trcp recognizes the phosphorylated residues Ser33 and Ser37, targeting β-catenin for ubiquitylation and proteasomal degradation. These regulatory phosphorylation sites are commonly mutated in liver cancer. [Casein Kinase 1 (CK1); Glycogen Synthase Kinase 3 (GSK3); β-transducin-repeat-containing protein (β-Trcp)]

In the Wnt “off” state, β-catenin is primarily involved in cell-cell adherens junctions and free cytoplasmic β-catenin is targeted for degradation by a cytoplasmic destruction complex that contains Axin, adenomatous polyposis coli (APC), glycogen synthase kinase3-β (GSK3β) and casein kinase 1 (CK1). Sequential phosphorylation of the N-terminus of β-catenin by CK1 and GSK3 leads to recognition of β-catenin by the E3 ubiquitin ligase β-transducin repeat-containing protein (β-trcp). Ubiquitylation of β-catenin targets it for proteasomal degradation and keeps cytoplasmic and nuclear levels of β-catenin low (Fig. 3) [22]. In the “on” state, Wnt binds to FZD and its co-receptor low-density lipoprotein receptor-related protein (LRP)-5/6 to selectively activate β-catenin-mediated canonical Wnt signalling. This triggers a series of down-stream events that culminates in the inhibition of the destruction complex, non-phosphorylated β-catenin accumulates in the cytoplasm, translocates to the nucleus and forms a transcriptionally active complex with the T-cell-specific transcription factor/lymphoid enhancer-binding factor (TCF/LEF) to initiate the expression of Wnt target genes (Fig. 2). The canonical Wnt/β-catenin pathway is regulated by many naturally occurring antagonists that act to inhibit Wnt binding to FZD [for example, secreted FZD-related protein (sFRP) and Wnt inhibitory factor (WIF)] or FZD binding to LRP [for example, Dickkpfs (DKK)] (Fig. 2) [23].

Wnt signalling is involved in several physiological and pathophysiological processes during embryonic development and carcinogenesis [18, 24]. Wnt/FZD/β-catenin signalling plays a critical role in liver development, liver regeneration and liver zonation which is required for spatial separation of the diverse metabolic functions performed in the liver [24]. Aberrant and constitutive activation of the Wnt pathway leads to uncontrolled cell proliferation and survival, promoting the development of several cancers including HCC [24] and HBV associated HCC [25]. In HCC, β-catenin accumulation has been observed in up to 50% of tumours and nuclear accumulation has been correlated with tumour progression and poor prognosis [26]. Mutations in β-catenin gene 1 (*CTNNB1*) that constitutively activate Wnt signalling have been reported in up to 40% of HCC cases [27]. The mutations at the N-terminus of the gene remove the GSK3β/CK1 phosphorylation sites, that normally target β-catenin for proteasomal degradation and leads to its stabilisation and translocation to the nucleus (Figs. 2 and 3). Further regulation of the Wnt pathway in HCC occurs via silencing through promoter hypermethylation of genes that code for the naturally occurring inhibitors of Wnt-FZD-LRP interaction. Hypermethylation of inhibitors of the receptor ligand complex are a common feature of Wnt-addicted cancers, irrespective of mutations to down-stream components of the pathway that constitutively activate Wnt signalling [23].

The Wnt/FZD/β-catenin signalling pathway is also linked to cell cycle regulation [28] and has a crucial role in the over expression of cyclin B1, C and D in the development of HCC [29, 30]. Furthermore, the Wnt/β-catenin signalling pathway interacts with other pathways that are deregulated in HCC including the Ras/ERK, PI3K/Akt/mTOR, and the IN/IRS1/IGF pathways (Fig. 1) [6]. These observations are significant in light of the previous reports showing that HBV infection is accompanied by the over expression of both cyclins B1 and D, the upregulation of PI3K/Akt and the inactivation of GSK3β [8–10] and the Wnt/β-catenin signalling pathway as a central component of HBV associated cell signalling events [6].

With the important role of Wnt/FZD/β-catenin signalling in the development of HCC, and other cancers, intense research efforts have been directed to developing new compounds to inhibit this pathway [26]. Inhibitors of the Wnt/FZD/β-catenin pathway (such as CGP049090, PKF115–584 and PKF118–310) have been shown to inhibit tumour growth in a number of HCC cell lines in culture [31]. These compounds in combination with other effective molecules result in a significant improvement in survival of patients with HCC [3]. A critical question to address is the role these treatment combinations have in patients with HBV associated HCC and their effect on HBV replication in hepatocytes.

#### PI3K/AKT and Ras/ERK1/2 pathways

Infection of hepatocytes with HBV is associated with the upregulation of PI3K/AKT and Ras/ERK1/2 [8–10] (Fig. 1). The consequences of this are a myriad of interrelated downstream effects that alter cell proliferation, cell cycle, apoptosis and ultimately contribute to oncogenic transformation. The up-regulation of both PI3K/AKT and Ras/ERK1/2 signalling pathways have been shown in both preneoplastic liver foci and in HCC’s [32]. The activity of these kinases is closely related to the regulation of p21^cip1^, which is increased in HBV infection of hepatocytes and contributes to G2 cell cycle arrest [8–10].

The PI3K/AKT pathway has a pivotal role in cell proliferation and the up-regulation of AKT results in the over-expression of cyclin D1, which has also been linked to HCC development [30]. Once activated AKT phosphorylates and inhibits GSK3β and enhances cell survival and proliferation, activates c-Myc and NFκB [8–10, 33, 34]. Inactivation of GSK3β is central to several signalling pathways and leads to the subsequent degradation of cyclin D1, c-Myc and β-catenin thereby contributing to the over expression of these proteins and to HCC development [18, 24]. The Ras-ERK signalling pathway also plays a central role in regulating the growth and survival of cells [34], is activated by HBV and is intricately linked with the cell cycle machinery through the activation of cyclin D1 [8, 34]. The changes that occur within these pathways as a consequence of HBV infections are closely interrelated to regulate a fine balance that ultimately determines cell proliferation, viral replication, cell survival or death and ultimately oncogenic transformation.

The PI3K/AKT pathway also has a pivotal role in cell proliferation and the up-regulation of AKT results in the over-expression of cyclin D1, which has also been linked to HCC development [35]. A coordinated upregulation of both AKT and ERK is required for optimal activation of cyclin D1 [33, 36]. Once activated AKT enhances cell survival and proliferation, activates c-Myc and NFκB [33, 36] and has the important role of inhibiting GSK3β [34, 37]. Inactivation of GSK3β by pAKT prevents the phosphorylation and subsequent degradation of cyclin D1 and c-Myc and thereby contributes to the over expression of these proteins.

The Ras-ERK signalling pathway also plays a central role in regulating the growth and survival of cells [34] and is intricately linked with the cell cycle machinery through the activation of cyclin D1 and the cyclin dependent kinases CDK4 and 6 together with the activation of cyclin E/CDK2 and c-Myc [34]. In addition, pERK activates JAK/STAT signalling resulting in the upregulation of STAT3, which has been linked to oncogenic transformation [38].

Finally, the upregulation of c-Myc protein by HBV [8] would also be expected to contribute to HBV oncogenesis [33, 36] as this factor has an important role in concomitantly inducing both cell proliferation and apoptosis (in a p53 dependent manner) as a mechanism to protect against the selection of proliferative cellular lesions that might result in unrestrained cell growth.

The changes that occur within these pathways as a consequence of HBV infection are not isolated events but closely interrelated to regulate a fine balance that ultimately determines cell proliferation, viral replication and cell survival or death. It is these many and varied effects that determine cell fate and also place the PI3K/AKT pathway at the centre of the mechanisms that underlie HBV associated hepatocarcinogenesis.

### HBV, hepatocyte apoptosis and HCC

Hepatocytes are particularly susceptible to Fas Ligand (FasL) induced apoptosis [39]. FasL is a member of the tumour necrosis factor (TNF) superfamily playing well-defined roles in the regulation of the immune system, embryonic development and tissue homeostasis. During acute HBV infection, liver-infiltrating lymphocytes will expose hepatocytes to FasL and induce widespread cell death [40]. While mature hepatocytes are highly sensitive to FasL, they are resistant to other death ligands such as TNF or TRAIL [41]. However, upon HBV infection, hepatocytes also become susceptible to these death ligands [42]. This sensitisation to death stimuli may be an important contributor to the extensive liver destruction that can follow hepatitis B infection. It has been shown that replicating HBV causes hepatocyte apoptosis [43–46]. Interestingly, reports using transgenic mice containing the whole HBV genome have shown that enhanced hepatocarcinogenesis is associated with increased apoptosis and compensatory regeneration [47, 48].

Human data show that HBV infected patients, particularly those with detectable serum HBV surface antigen (HBsAg), are at increased risk of HBV reactivation when treated with TNF antagonists. Hepatocyte apoptosis mediated by TNF is an important mechanism by which HBV infected cells are eliminated from the liver. Therefore, therapeutics that augment the mechanisms through which TNF constrains HBV could be of great benefit to patients with chronic HBV infection. Cellular inhibitor of apoptosis proteins (cIAPs) regulate TNF signalling by promoting NF-κB activation downstream of TNF receptor 1 (TNFR1) ligation, and this activation, in turn, promotes cell survival by antagonizing TNF mediated cell death. By attenuating TNF signalling during hepatitis B infection, cIAPs thereby restrict hepatocyte death and allow viral persistence. However, when the function of cIAPs is antagonized, TNF-mediated ligation of TNFR1 causes cell death. Mice deficient in cIAPs in the liver have an enhanced clearance of HBV infection [46]. This major finding raises the possibility of targeting IAPs to promote HBV clearance in patients. By inhibiting cIAPs with SMAC mimetics HBV can be eliminated from infected livers [45]. This provides an important therapeutic approach for the treatment of chronic hepatitis B and of HBV associated HCC. It is possible that combination therapy with inhibitors of the Ras/ERK, PI3K/AKT/mTOR and Wnt/β-catenin together with SMAC mimetics could result in an enhanced anti-tumour effect and increased viral clearance [45].

### HBV, cell cycle and HCC

The cellular mechanism underlying the development of hepatocellular carcinoma in HBV infected patients is likely to be multifactorial. An initiating oncogenic stimulus, which results in a number of changes within cell signalling pathways and cell cycle regulation, is tightly linked with an inflammatory and cytokine reaction driven by Kupffer cells in response to degradation products of apoptotic cells, chemical carcinogens and viral antigens [5, 13–15].

Like many DNA viruses, HBV manipulates the cell cycle machinery so that it can replicate more efficiently. Two important components of cell cycle regulation that are affected by HBV include a member of the kinase inhibitor protein family, p21^cip1^ [8–10, 49–52], which mediates cell cycle arrest at the G1/S and G2/M boundaries of cell cycle by both p53-dependent [49, 51] and independent [50, 52] mechanisms and the Wnt/β-catenin pathway [27]. Activation of ERK has been proposed to underlie the intranuclear accumulation of p21^cip1^ in G2 which appears to serve an important role in delaying cell cycle progression to mitosis in order to protect cells against DNA damaging agents and apoptosis [50, 52]. HBV infection of hepatocytes is associated with the activation of ERK and the subsequent up-regulation of p21^cip1^thereby producing G2 cell cycle arrest [8–10]. This arrest serves a beneficial role for the virus because viral replication is increased in both G1 and G2 phases of cell cycle [53]. These studies serve to further reinforce the importance of elucidating the relationship between HBV and p21^cip1^ and apoptotic responses in hepatocytes.

The Wnt/β-catenin pathway has also been shown to regulate G2 cell cycle arrest [54]. Cell cycle arrest may be beneficial for HBV because it results in enhanced viral replication in both G1 and G2 phases of cell cycle. The reliance of HBV on cell cycle and signalling events means that modulation of these cellular events with small molecules could result in viral clearance. HBV modulates the regulation of cellular transcription to promote cell proliferation, cell growth, cell survival and cell metabolism, [11, 55, 56]. Furthermore, HBV has been shown to promote HCC development by modulating the Wnt/β-catenin pathway [57]. It has also been demonstrated that HBV, through the inhibition of the Smc5/6 complex, is a critical regulator of cellular chromatin and this may thereby regulate HBV cccDNA transcriptional activity [58]. The role of Wnt/β-catenin signalling on the Smc5/6 complex and HBV cccDNA transcriptional activity has not yet been determined and warrants further investigation.

### Limitations in the study of HBV associated HCC: benefits of liver organoids

**Fig. 4:**
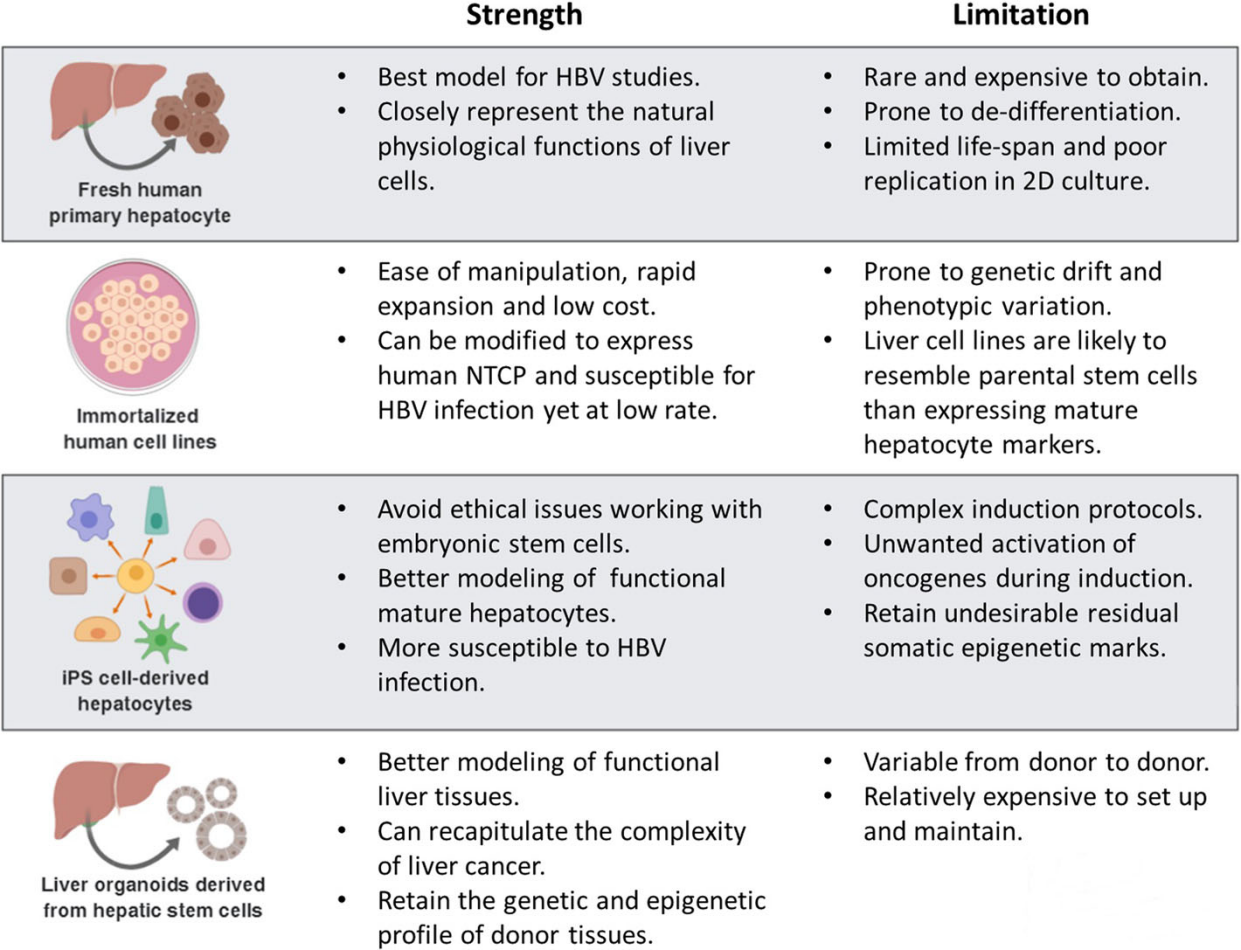
In vitro models for studying HBV infection*.* Primary human hepatocytes derived from liver tissue provide the best material for HBV studies; however, human liver tissue is not readily available and is expensive to source and process. However, the discovery of human NTCP as one of the membrane receptors for HBV binding has allowed for the development of immortalized cell lines susceptible to HBV infection. iPS technology has helped to create better models that resemble functional mature hepatocytes and yield better HBV infection. But these two in vitro models still have several limitations, especially in regard to the genetic and epigenetic profiles of cells arising from different individual sources. Recently, a newly developed technique allows for the production of liver organoids directly from hepatic stem cells in liver tissue, creating a superior model for future HBV studies

**Fig. 5:**
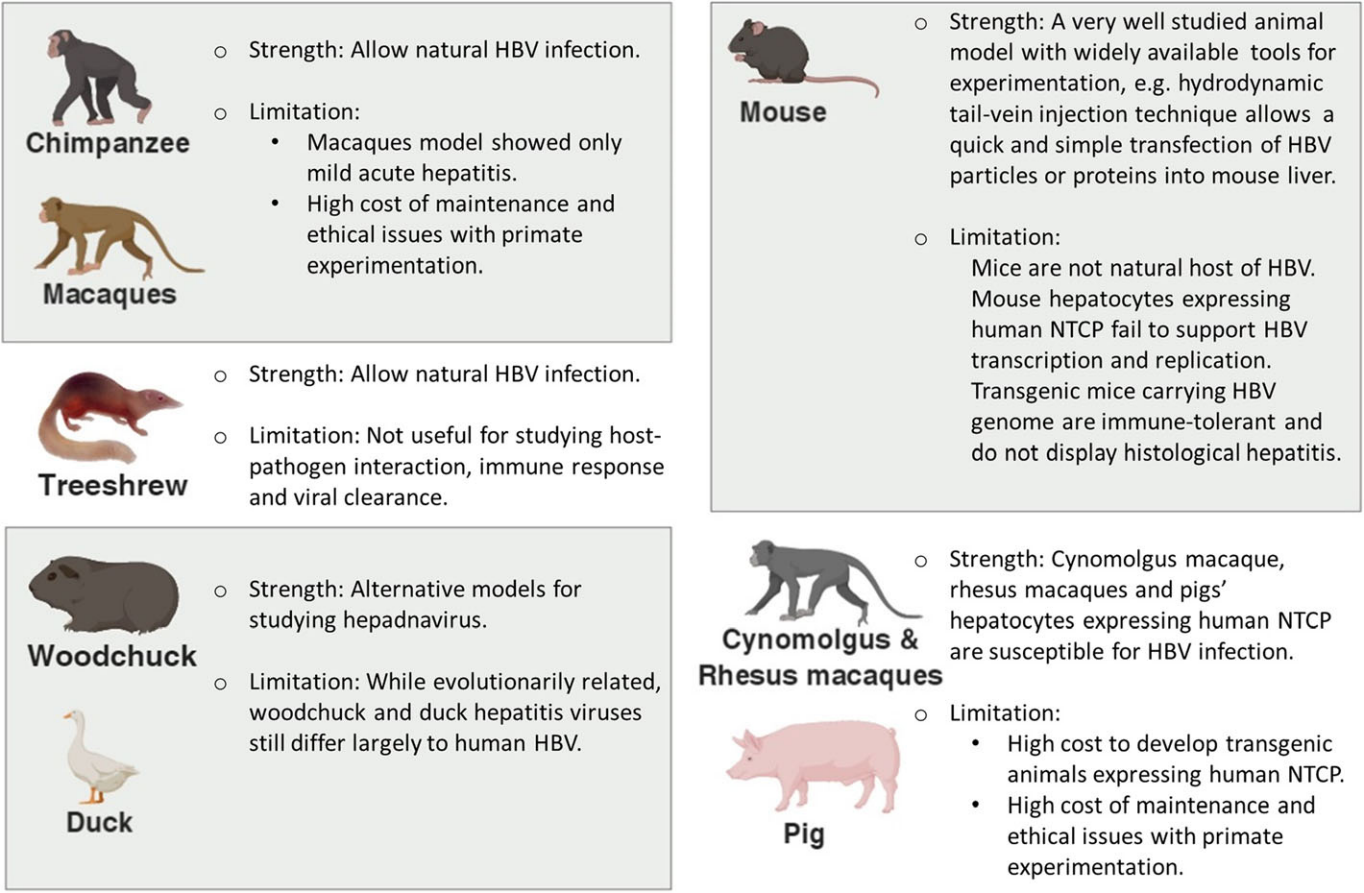
Animal models for studying HBV infection. Primates are the best models for studying HBV infection, but the associated high cost and regulations with animal ethics present significant limitations in the use of primates for future research. Alternative models using treeshrew, woodchuck, duck and mouse are useful, but these models are limited in progressing studies on host-pathogen interactions, immune response and viral clearance in humans. New potential models using transgenic macaques or pigs expressing human NTCP may help bridge this gap

Although HBV is a known hepatocarcinogen, the precise mechanism by which it causes HCC is unknown. An important obstacle to the study of molecular mechanisms underlying HBV associated HCC development has been the lack of in vitro (Fig. 4) and in vivo (Fig. 5) model systems that support human HBV infection. This is primarily because HBV is a hepatotropic virus that only infects human hepatocytes.

HCC is a complex multistage and multifactorial disease. The molecular pathogenesis and host-viral interactions that drive tumourigenesis remain elusive. One of the main challenges is the lack of satisfactory model systems to elucidate the underlying mechanisms. At the same time, there are major unmet needs for tumour characterisation and personalised therapeutic strategies to target driver mutations for better treatment outcome. A wide range of infection models have been used to address these unmet needs.

#### Fresh human primary hepatocytes

A lack of suitable human liver models for both HBV and HCV has hampered research in to the pathogenesis and developing a cure for HBV and vaccine development for HCV. Although the best source of primary human hepatocytes is fresh resected liver [59, 60] these cells are prone to de-differentiation, gradually losing their hepatic functionality [61]. This reduces the infectivity of primary hepatocytes by hepatitis viruses [62, 63]. In addition, these cells appear to have a limited lifespan and replicate poorly in 2D cultures (1 week) and sandwich cultures (2 weeks) [64–66] (Fig. 4).

#### Immortalized human cell lines

Immortalized continuous liver cancer derived cell lines have been the preferred model system to overcome the limitations of accessing primary human hepatocytes. These cell lines have been crucial to date for both research for pharmacological drug screening and validation (Fig. 4). The main strengths of continuous cell lines include ease of genetic manipulation, rapid expansion at comparatively low maintenance costs and thorough characterisation. However, it is well-known that continuous cell lines cultured in vitro are prone to genetic drift [67], or displaying phenotypic variation [66, 68, 69]. This could partially explain why there is no correlation between genetic expression patterns for multi-drug resistance observed when cell lines were compared to clinical primary cultures [70]. Interestingly, immortal cell lines, even though derived from different cancer types, are more likely to resemble each other rather than the clinical samples they were supposed to model [70–72]. Most of the routinely used liver cancer cell lines e.g. Huh7 and HepG2 not only show different morphology between laboratories but also a downregulation of mature hepatocyte markers such as albumin or cytochrome P450 (CYP) family. Furthermore, none of the cell lines support natural HBV infection, probably due to the lack of mature hepatocyte receptors necessary for viral entry. Recently, sodium taurocholate cotransporting polypeptide (NTCP) was described as a putative receptor required for HBV entry and infection [73]. Since then, many attempts to introduce the NTCP transgene into hepatoma cell lines were made but, unlike natural infection, resulted in low levels of infection [73] and poor viral spreading [74]. In addition, while the expression of human NTCP confers susceptibility to HBV infection, continuous cell lines such as Huh7, HepG2 and HepaRG show a broad range of differences in susceptibility for HBV [75] as well as viral DNA integration [76]. This evidence suggests that HBV infectivity is not only determined by the binding receptor, but also through subsequent post-binding events or cell surface receptors in addition to NTCP. Recently, a newly developed hepatoma cell line named HLCZ01 that supports HBV infection by patient sera has been described [77], opening a new promising prospect for HBV research.

Attempts have also been made to create immortal continuous human hepatocyte cell lines using viral oncogenes to bypass growth arrest of cultured adult hepatocytes, such as transduction of the simian virus *40 T antigen* (SV40 Tag) or human papillomavirus 16 *E6/E7* genes [78–82]. Human hepatocytes are also known to possess limited in vitro proliferative capacity due to the restriction in their telomerase activity. Therefore, they are prone to replicative senescence [83, 84]. This problem can be overcome by transfecting *hTERT* retrovirus or Cre-loxP with the tet-on and -off system [81, 85]. However, immortalisation using *hTERT* is only suitable for a small subset of human hepatic cells, including foetal and neonatal hepatocytes, with mixed results [86–88].

#### Induced-pluripotent stem cell-derived hepatocytes

Since the discovery of “Yamanaka” factors (Oct3/4, Sox2, Klf4, and c-Myc) [89], there has been huge interest in induced-pluripotent stem (iPS) cell technology. Apart from avoiding ethical issues in working with stem cells, iPS cells bring a far-reaching promising impact for biomedical research, especially for tissue engineering, personalised medicine, disease modelling, and transplantation research (Fig. 4). One of the advantages of iPS cells is their ability to generate pluripotent stem cells from any cell source and can differentiate into any cell type. However, iPS cells possess a number of limitations [90]. iPS technology relies on using retrovirus or lentivirus for effective delivery of “Yamanaka” factors into somatic cells, hence posing the grave risk of unwanted activation of oncogenes or disrupting coding sequences of certain regulatory genes due to the random integration of viral vector into host transformed cell genome. Moreover, there is a high risk of ectopic expression of exogenous Oct3/4, Sox2, Klf4, and c-Myc genes inside iPS cells [91] that renders the cell lines unsuitable for transplantation due to high risk of neoplastic transformation. In fact, expression of this set of genes was found to be associated with tumourigenesis in a clinical setting [92]. Also, overexpression of Oct4 alone can cause epithelial cell dysplasia in mice [93], ectopic expression of Sox2 is associated with mucinous colon carcinomas [94], while Klf4 is linked to breast cancer [95]. C-Myc is itself found to be overexpressed in more than 70% of cancers [96]. Indeed, in iPS cells, c-Myc reactivation was often found to be associated with tumour development [97, 98].

Many alternative technologies have been developed to address these safety concerns. On one hand, mouse iPS cells were generated free from viral vectors through repeated transfection of expression plasmids containing cDNAs for the “Yamanaka” factors [97]. On the other hand, there have been many attempts to limit the use of c-Myc in mouse and human fibroblasts [99, 100]. With respect to modelling functional liver tissue, iPS cells can generate liver buds and be successfully implanted to rescue liver failure in mice [101]. However, the majority of iPS cell-derived human hepatocytes are still inferior to primary hepatocytes isolated from resected liver. Rather, iPS-derived hepatocytes mimicked foetal hepatocytes [102–104]. One reason for a lack of adult features may be due to the appropriate stoichiometry of reprogramming factors as well as the origin of the transformed cells required for proper reprogramming. Additionally, for iPS cells to be fully differentiated into mature hepatocytes, suitable epigenetic modifications must occur [105], as some human iPS cells tend to retain residual somatic epigenetic markers, leading to certain DNA methylation profiles [106]. For instance, a recent study showed that the CYP enzyme promoter in embryonic stem cell-derived hepatocytes is highly hypermethylated, leading to an inferior CYP profile compared to primary hepatocytes [107]. Experimentally, inhibition of DNA methyltransferases and histone deacetylases in this cell line can rescue this phenotype, but this is a real limitation for application in a clinical setting.

In addition, differentiation of iPS-derived hepatocytes also depends heavily on the induction cocktail [108] as well as stromal, endothelial [109, 110], fibroblast [111], and hepatic resident cells that help ameliorate the differentiation efficiency [101]. In fact, even primary hepatocytes co-cultured in a specific pattern with stromal cells to better mimic the in vivo situation illustrated higher NTCP expression and supported better HBV infection [112]. Recent studies show that NTCP expression in differentiated iPS cells can achieve a level close to primary hepatocytes; however, the infection efficiency is still very modest [113, 114]. In addition, efficient viral infection required a very high multiplicity of infection (MOI). An MOI of 50 gave 30% HBV-positive cells while an MOI of 200 yielded 60% infected cells and MOI of 1000 resulted in infection of almost every cell [115]. Another study using iPS-derived hepatocytes grown in a 3D culture system also showed a similar result [116]. This strongly suggests that the HBV infection may require additional elements besides NTCP.

#### In vivo animal models

Among animals available for HBV research, the chimpanzee is the only model that can be infected naturally by patient sera and develop chronic infection closely mimicking human conditions (Fig. 5). However, due to substantial ethical concerns, the use of chimpanzees for HBV research is heavily restricted. Other primate models, such as gibbons or macaques, can be infected with HBV; however, they showed only a mild acute hepatitis. Hence, alternative animal models such as woodchuck or duck have been used to study hepadnavirus virology, as the respective infectious agents are evolutionarily related to human HBV. Yet, these animal hepatitis viruses still differ extensively from HBV. Recently, a macaque species named *Macaca fascicularis* from Mauritius Island [117] was found to support natural HBV infection and develop chronic infection. However, a number of ethical issues working with these primates still exist.

For non-primate animals, HBV can only infect tree shrew tupaia [118]. Many attempts were made to develop a mouse model that is susceptible to HBV infection. In fact, transgenic mouse models, although not entirely mimicking the scenario of HBV infection in humans, have provided some meaningful data for better understanding of the replication and life cycle of HBV. Also, mice expressing human NTCP fail to support natural HBV infection, but support hepatitis D virus which exploits HBV surface proteins to initiate infection [75]. In particular, it was shown that HBV could gain entry into human NTCP-expressing mouse hepatocytes, but failed to carry out viral transcription [119] due to the lack of a host cell dependency factor [120]. Other approaches include using human liver transplantation into mice to develop chimeric humanised mouse models (reviewed in [121]) which are susceptible to natural HBV infection. However, the process is very complicated and expensive.

A full-length HBV genome transgenic mouse has also been shown to produce complete infectious HBV particles [122]. However, because these mice are immune-tolerant to HBV, they do not show histological changes in the liver consistent with hepatitis. Similar approaches to create transgenic mice expressing HBV structural proteins were also carried out for surface proteins [123], precore and core [124], and HBx [125]. Among these studies, only HBx transgenic mice developed HCC. However, later studies disputed this claim, referring to the functional role of HBx only as a potential oncogenic agent [126, 127].

Although HBV transgenic mouse models are very valuable, they are not very useful to investigate host-pathogen interactions, especially with respect to the immunological response and viral clearance. That is not to mention the labour, cost, and time to generate and validate the models. In fact, the most efficient mouse model for studying the immunopathogenesis of HBV infection makes use of hydrodynamic tail-vein injection to introduce HBV into mice. This technique results in a high efficiency of transfection of hepatocytes [128, 129]. Hence, the in vitro studies of HBV proteins could be translated into in vivo models easily. The main benefit of this technique over in vitro models is for studying the immune response in scenarios of acute infection as well as host-pathogen interaction of HBV proteins [130].

Recently, it was shown that complementation of primary hepatocytes isolated from cynomolgus macaques, rhesus macaques, and pigs with human NTCP by adenoviral transduction resulted in fully susceptible HBV infection comparable to human hepatocytes [131]. However, like mouse models, the process to create human-NTCP transgenic animals from these species for in vivo studies is very complicated and expensive and introduces additional ethical issues in working with these animals.

#### Liver organoid derived from adult hepatic stem cells

Recently, Huch and colleagues [132, 133] showed that leucine rich repeat containing G protein-coupled receptor 5 (LGR5) expressing cells isolated from liver tissue can give rise to 3-dimensional ex vivo mini-liver structures, referred to as liver organoids (Fig. 4). These liver organoids not only retain the characteristics of their original tissue, they are also able to undergo unlimited expansion and can be differentiated into mature hepatocyte-like cells that can rescue a liver-defective mouse model [132]. Thus, this provides a very valuable cell source for disease modelling, toxicology testing, as well as transplantation research and for personalised medicine.

Compared to iPS-derived hepatocytes, the adult stem-cell-derived liver organoid model possesses many advantages. Firstly, liver organoid cells have been shown to have a stable genome and very low risk of spontaneous mutation or chromosomal alteration [133]. Secondly, as they originate from liver tissue, they are not subjected to unexpected hypermethylation of liver-specific markers or functional proteins, which is common in iPS generated organoids. In addition, the hepatic stem cells that give rise to liver organoids are relatively tough and resilient. Primary tissues stored in cold medium for 3 days, or frozen in DMSO or snap-frozen in liquid nitrogen can still yield organoids [134]. The same properties have not been observed in iPS-derived liver cultures. Another advantage of liver organoids is the straightforward and robust protocol for isolating and expanding cells from donors’ tissue. Established organoids can be cryopreserved and recovered, which is very important for tissue banking. One of the reasons for these advantages is due to the 3D Matrigel culture that allows hepatocyte polarisation as well as facilitating interactions between cell-cell and cell-microenvironment [135]. In fact, hepatic stem cells cultured on a 2D monolayer fail to maintain hepatic stemness [136] and the cellular chromosomes become unstable after long-term passage [137]. Adult tissue stem-cell-derived organoids in 3D culture helps bridge this gap [138]. HBV only infects mature human hepatocytes. Hence, the differentiated liver organoids are susceptible to natural infection with HBV, producing high titre virus in the supernatant [139]. The first report of human liver organoid culture and characterisation was in 2015 [133], and this year, the first publication on HBV infection of these organoids [139]. Much has been learnt from iPS and the hepatoma HLCZ01, thus the development of a liver organoid model system susceptible for HBV infection was much easier.

While iPS cells and hepatoma cell lines stop short as a model system for researching HBV infection or drug assay for anti-viral capability, liver organoids offer far more scope. For example, HBV chronic-infected patients who are at high risk for developing cirrhosis or HCC, liver organoid technology can support the development of promising personalised medicine regimens including but not limited to: testing cirrhosis/tumour tissues for precise drug regimes, manufacture potential materials with high susceptibility for transplantation, or generate genome-edited ex vivo tissue free of defects or with boosted immunological response. This is especially important for HCC, where tumour tissues are extensively heterogeneous and only the liver organoid system can closely recapitulate this complexity. Thus, it is tremendously valuable for better diagnosis and treatment. In fact, many translational applications along these lines have been carried out for pancreatic [140] and colorectal cancer [141]. None of this can be achieved using iPS cells, in vivo mouse models or immortal cell lines (Figs. 4 and 5).

## Conclusions

The organoid revolution led by the Clevers laboratory has made personalised medicine a tangible reality for solid tumours of the colon, breast and pancreas. Using high throughput screening, tumour cell responses can be tested to drugs in isolation and in combination. Our ability to now grow HCC tumours in tissue culture as patient-derived tumour organoids means we can do the same for liver cancer patients. Furthermore, organoid technology has led to a better understanding of the molecular mechanisms of how a normal colon epithelial cell becomes a cancer cell. We are on the cusp of extending this understanding to liver cancer to curtail the alarming trend in mortality.

On the other hand, organoids established from normal human tissues are proving to be invaluable models of natural infection, particularly for pathogens that only infect human cells. Normal liver organoids were first established, again by the Clevers laboratory, in 2013 [132], and the further improvements reported recently by the group [142] will no doubt advance our understanding of the oncogenic interplay between HBV infection and cellular signalling pathways.

## Data Availability

Not applicable. We do not include any data.

## Abbreviations

3D: 3 dimensional
AIDS: Acquired Immune Deficiency Syndrome
APC: Adenomatous polyposis coli
cIAPs: cellular inhibitor of apoptosis proteins
CK1: Casein kinase 1
CYP: Cytochrome P450
DKK: Dickkpfs
DMSO: Dimethyl sulphoxide
FasL: Fas Ligand
FZD: Frizzled
GSK3β: Glycogen synthase kinase3-β
HBsAg: HBV surface antigen
HBV: Hepatitis B Virus
HBx: HBV X protein
HCC: Hepatocellular Carcinoma
HIV: Human Immunodeficiency Virus
IGF: Insulin-like growth factor 1
iPS: induced-pluripotent stem
IRS1: Insulin receptor substrate 1
JNK: c-Jun N-terminal kinase
LEF: Lymphoid enhancer-binding factor
LGR5: Leucine rich repeat containing G protein-coupled receptor 5
LRP: Low-density lipoprotein receptor-related protein
MAPK: Mitogen-activated protein kinases
MOI: Multiplicity of infection
mTOR: mammalian target of rapamycin
NTCP: Sodium taurocholate cotransporting polypeptide
PI3K: Phosphatidylinositol-4,5-bisphosphate 3-kinase
sFRP: secreted FZD-related protein
SOCS3: Suppressor of cytokine signalling 3
TCF: T-cell-specific transcription factor
TNF: Tumour necrosis factor
TNFR1: TNF receptor 1
WIF: Wnt inhibitory factor
β-trcp: β-transducin repeat-containing protein
